# Control of Chromatin Organization and Chromosome Behavior during the Cell Cycle through Phase Separation

**DOI:** 10.3390/ijms222212271

**Published:** 2021-11-12

**Authors:** Jiaxiang Li, Jinmin Gao, Ruoxi Wang

**Affiliations:** Key Laboratory of Animal Resistance Biology of Shandong Province, Institute of Biomedical Sciences, College of Life Sciences, Shandong Normal University, Jinan 250014, China; 17853136665@163.com (J.L.); jinmingao@sdnu.edu.cn (J.G.)

**Keywords:** liquid-liquid phase separation (LLPS), chromosome organization, mitosis, meiosis

## Abstract

Phase-separated condensates participate in various biological activities. Liquid–liquid phase separation (LLPS) can be driven by collective interactions between multivalent and intrinsically disordered proteins. The manner in which chromatin—with various morphologies and activities—is organized in a complex and small nucleus still remains to be fully determined. Recent findings support the claim that phase separation is involved in the regulation of chromatin organization and chromosome behavior. Moreover, phase separation also influences key events during mitosis and meiosis. This review elaborately dissects how phase separation regulates chromatin and chromosome organization and controls mitotic and meiotic chromosome behavior.

## 1. Introduction

During eukaryotic development, highly ordered cellular activities are controlled by membrane-enclosed organelles, as well as membraneless compartments. The molecular mechanisms underlying the dynamics of membrane-enclosed organelles, such as their fusion and fission, vesicle-mediated trafficking, and membrane contact-mediated interactions between organelles, have been extensively described. However, the molecular mechanisms and functional studies of membraneless organelle assembly are yet to be explored. A growing body of experimental evidence has found that the formation and development of such membraneless structures can be explained in terms of phase separation. Liquid–liquid phase separation, driven by collective interactions between multivalent and intrinsically disordered proteins, is thought to mediate the formation of membraneless organelles in cells [1]. Biomacromolecules, such as proteins and nucleic acids, can be coalesced by LLPS into liquid-like, membraneless condensates that organize intracellular components into multiple compartments and thus perform different biological functions [2]. Experiments involving fluorescence recovery after photobleaching that were used to ascertain the impact of LLPS on aggregation kinetics have demonstrated that protein aggregation occurs both within and outside the context of LLPS [3]. In addition, membrane-free compartments of the same type fuse with one another when contact occurs [4,5]. These phenomena suggest that phase-separated structures have dynamic properties.

LLPS also plays an important role in the cell cycle. Phase-separated condensates regulate chromatin organization and chromatin behavior during the cell cycle. For example, the chromosomal passenger complex (CPC) abundantly localizes to inner centromeres, a region on mitotic chromosomes that enables specific biochemical reactions. The CPC, recruited through phase separation, works together with aurora kinase activity to regulate chromosome segregation during mitosis [6]. In addition, the mechanisms of many key events during meiosis remain poorly understood, and accumulating evidence suggests that multiple meiotic events might be regulated by phase separation. For instance, in *Schizosaccharomyces pombe* meiosis, lncRNA–protein complexes form condensates at specific chromosomal loci to mediate the recognition and subsequent pairing of homologous chromosomes [7]. In *Caenorhabditis elegans*, multivalent weak interactions between synaptonemal complex (SC) units drive SC formation, and the SC exhibits phase separation properties [8]. DNA-driven condensation assembles the meiotic DNA double-strand break machinery, the multilayer control of Spo11 resulting from the recruitment of regulatory components, and the modulation of the biophysical properties of the condensate [9]. In mammalian oocytes, a liquid-like spindle structural domain facilitates spindle assembly [10]. Increasing evidence highlights the involvement of phase separation in regulating chromatin assembly, as well as chromosome behavior. Herein, we review the recent advances in understanding the regulation of chromatin organization and chromosome behavior through phase separation during mitosis and meiosis.

## 2. Regulation of Chromatin Organization by Phase Separation

**Figure 1:**
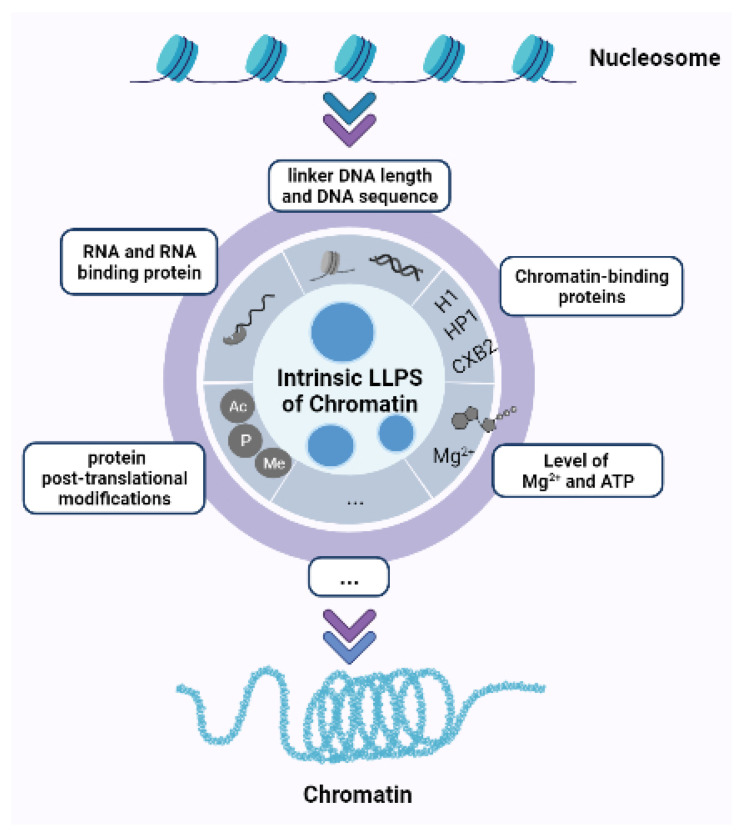
Regulation of chromatin organization by phase separation. Linker DNA length and DNA sequence specificity, chromatin-binding proteins (i.e., H1, HP1, and CXB2), level of Mg2+ and ATP, RNA and RNA binding protein, and protein post-translation modifications (i.e., phosphorylation, acetylation, and methylation) can all affect chromosome organization by different phase separation. There are still more factors affecting chromosome organization by phase separation to be explored in further studies.

In the nucleus of eukaryotic cells, genomic DNA is highly condensed by proteins into nucleoprotein complexes called chromatin. The structure of chromatin plays a variety of roles in eukaryotes, from the structural organization of the genome to the facilitation of transcription factors and remodeling of individual gene promoters [11]. Higher-order chromatin structure typically refers to those structural features of the genome that serve to facilitate large-scale condensation and packaging [11], including topologically associating domains, loop, euchromatin, or heterochromatin. They play an important role in chromosome dynamics and genome integrity. More evidence indicates that nuclear chromatin can be liquid-like, and the phase separation model helps explain the observed chromatin organization patterns. In the following, we review recent advances detailing the factors that affect the phase separation of chromosome organization (Figure 1).

### 2.1. Linker DNA Length and DNA Sequence

The Rosen lab found that the lengths of internucleosomale linkers affect the LLPS of chromatin. In comparing chromatin with either long (10n + 5) or short (10n) nucleosome spacing, they found that the propensity to undergo phase separation was favored by long spacing and reduced by short spacing. These observations suggested that long spacing of nucleosomes is preferred in cells, which is beneficial for encoding the higher-order nucleosome organization inherent to chromatin [12]. Moreover, the Kim lab combined fluorescence resonance energy transfer measurements, precipitation assays, and molecular dynamics simulations and found that, DNA condensation depends not only on nucleotide composition but also on their sequence arrangement. Though coarse-grained simulations, it was found that TA-rich molecules spontaneously formed stable bundles, while CG-rich DNA remained dispersed or weakly bound to the periphery of the TA-rich clusters. This further implies that different affinities between DNA regions of different sequence patterns may drive chromatin phase separation into chromosomal subdomains [13]. In addition, the use of model analysis can improve our efficiency in understanding and predicting DNA behavior. For example, systematic multiscale coarse-grained simulation of DNA phase separation by the three-valent cobalt (III)-hexamine, allows the prediction of mesoscale levels of DNA condensation [14]. In the future, this approach may be generalized to model chromatin up to the size scale of whole chromosomes to help understand the regulation of chromatin organization.

It is generally accepted that important mechanisms of chromatin organization are explained by the DNA loop extrusion model [15]. Increasing experimental evidence suggests that phase separation is involved in chromatin organization. Fine-mapping of nuclear compartments using ultra-deep Hi-C showed that active promoter and enhancer elements are localized in the active A compartment, whereas the flanking sequences are comprised entirely of inactive chromatin and are localized in the B compartment. These results suggest that the DNA-binding regulatory complexes undergo phase separation at the scale of individual DNA elements [16]. Phase separation also drives aberrant chromatin looping and cancer development [17]. The intrinsically disordered regions (IDRs) of chimeric transcription factors induce DNA looping between super-enhancer-like targeting sites and oncogenes through phase separation [17]. Combinatorial interactions of chromatin factors, such as CTCF and cohesion, allow chromatin to self-assemble into segregated globules, which appears to be a robust chromatin organization mechanism with both stochastic and specific properties [18]. Chromatin folding means that the three-dimensional structure changes, which is usually accompanied by a change in cell fate. During this process, conserved topological-associated domains (TADs) are reorganized [19]. TAD reorganization-based multiomics analysis revealed that the dynamics of concentrated chromatin loops in octamer-binding transcription factor phase-separated condensates contribute to TAD reorganization, which promotes reprogramming. In addition, it is also popular to establish models to explain or predict chromatin DNA behavior. These pieces of evidence indicate the involvement of phase separation in the formation of chromatin domains.

### 2.2. Chromatin-Binding Proteins

Chromatin-binding proteins can regulate chromosome organization and chromosome condensation by phase separation. Histone H1 promotes the phase separation of chromatin, which increases the concentration of nucleosomes within droplets and decreases droplet dynamics, consistent with the role of histone H1 as a repressive chromatin architectural factor that promotes the condensation of chromatin in cells [12,20,21,22]. In vitro analysis showed that histone acetylation leads to chromatin droplets being dissolved, while highly acetylated chromatin can be re-induced to undergo phase separation by multi-bromodomain proteins, such as the transcriptional regulator BRD4 [12]. Interestingly, droplets formed by acetylated chromatin and multi-bromodomain proteins do not coalesce with droplets of non-acetylated chromatin [12]. The above observations suggest that chromatin polymers can undergo LLPS to generate physically adjacent but functionally distinct chromatin domains, which may play an important role in the organization and regulation of eukaryotic genomes [20].

Constitutive heterochromatin is an important component of eukaryotic genomes that has essential roles in heritable gene repression [23], the maintenance of chromosome integrity, and the provision of mechanical rigidity to the nucleus [24,25]. The core of a major heterochromatin type is the complex formed between heterochromatin protein 1α (HP1α) and chromatin in which histone H3 lysine 9 (H3K9me) is methylated. It has been found that the phosphorylation of the HP1α N-terminal region or DNA binding promotes the formation of phase-separated droplets, thereby chromatin condensation [25,26,27,28]. Moreover, the *Schizosaccharomyces pombe* HP1 protein Swi6 reshapes the nucleosome core, which increases opportunities for multivalent interactions between nucleosomes, thereby promoting phase separation [29]. These findings suggest that heterochromatin-mediated gene silencing may occur, in part, by sequestering compacted chromatin in phase-separated HP1α droplets.

Polycomb group (PcG) proteins are master regulators of development and differentiation. Mutations or dysregulation of the PcG genes can lead to developmental defects and cancer. PcG complexes, including Polycomb repressive complexes 1 and 2, act directly on specific chromatin regions to create Polycomb repressive domains. CBX2, a member of the CBX protein family, promotes the compaction function of PRC1 in mammals [30,31]. CBX2 mainly depends on phase separation to promote the formation of facultative heterochromatin [32]. In vitro, reversible electrostatic interactions between phosphorylated serine and positively charged residues within the CXB2 low-complexity disordered region are necessary for phase separation of CBX2. CBX2 undergoes phase separation when assembling Polycomb condensates onto chromatin by binding with DNA. This process accelerates the searching for cognate CBX2 binding sites on DNA, where the same or adjacent sites are repetitively revisited, thereby enhancing genomic occupancy by CBX2 [33].

### 2.3. Level of Mg^2+^ and ATP

Some monovalent and divalent cations affect the conformational and dynamic changes in chromatin. Monovalent cations can promote chromatin condensation at high concentrations but with limited ability. Divalent cations, such as Mg^2+^, affect phase separation and can influence the formation of higher structures of chromatin and chromosomes more effectively. Mg^2+^ induces sequence-dependent self-assembly of double-stranded DNA (dsDNA) and also promotes self-assembly between nucleosomes with identical DNA. Reducing electrostatic repulsion may be the principal role of Mg^2+^ ions in nucleosome self-assembly [34]. Interestingly, DNA methylation can also control cation-mediated DNA condensation [13]. Therefore, we speculate that Mg^2+^ and DNA methylation may have synergistic effects in regulating structural changes in chromatin. An explanation from the charge perspective is that ATP-chelated Mg^2+^ is hydrolyzed at mid-cytokinesis, and the concentration of free Mg^2+^ in the cell is thus elevated, which neutralizes the DNA charge, prompting chromosome condensation and, eventually, chromosome stabilization [35]. The presence of highly negatively charged hydrophobic residue can facilitate and drive phase separation [36]. In addition, an increase in ATP levels, which corresponds to a decrease in Mg^2+^, promotes the formation of phase-separated condensates [37]. ATP acts as a hydrotrope to maintain the solubility of macromolecules in the phase-separated droplets [38]. Mg^2+^ and ATP thus coordinate with one another to regulate the reversibility of phase separation. Moreover, ATP can also change the equilibrium thermodynamics of phase separation by leading non-equilibrium or non-thermal activity in the nucleus. For example, the dynamics of B cell chromatin loops also require a continuous ATP supply [39]. The binding of ATP enhances the thermodynamic stability of TDP-43 RRM domains, and ATP generally safeguards functional phase separation from transforming into pathological aggregation/fibrillation associated with various diseases and aging [40]. In summary, Mg^2+^ affects chromatin assembly by changing the DNA conformation mainly through the action of electric charge, while the ATP supply can control the occurrence of partial phase separation.

### 2.4. RNA and RNA Binding Protein

Phase separation of RNA-binding proteins shape large-scale chromatin structure and thus regulate genome function. Scaffold attachment factor A (SAF-A) can regulate interphase chromosome structure through interaction with nuclear RNA [41]. SAF-A interacts with nuclear RNA through phase separation to form a dynamic, transcription-responsive chromatin network that organizes the large-scale chromosome structure and protects the genome from instability [41,42,43]. Moreover, scaffold attachment factor B (SAFB) interacts via its R/G-rich region with heterochromatin-associated repeat transcripts, such as major satellite RNAs, which promote phase separation driven by SAFB, resulting in stabilized pericentromeric heterochromatin and subsequently maintaining the 3D genome structure [44].

Based on the CasDrop method developed with CRISPR/Cas-9, Shin et al. studied the nuclear condensation process for the LLPS of many intrinsically disordered proteins (IDPs). It is believed that nuclear condensates formed by phase separation may function as mechano-active chromatin filters that physically pull in targeted genomic loci, while pushing out non-targeted regions of the neighboring genome [45].

### 2.5. Protein Post-Translation Modifications

Protein post-translation modifications (PTMs) also modulate chromosome organization through phase separation. “Writer” and “eraser” enzymes for PTMs spatiotemporally modulate the propensity of IDR-containing proteins to undergo LLPS, which impacts membraneless organelle formation. Acetylation of histone tails antagonizes chromatin phase separation, dissolving droplets in vitro and decreasing droplet formation in nuclei [46]. This suggests that the acetylation of histone tails can cause chromatin decompaction in cells and is associated with open chromatin. However, it is still unclear how the LLPS propensity of different proteins is altered by acetylation. Phosphorylation at serine residues of the H1 C-terminal tail dramatically alters the higher-order structure in the condensates and reduces partitioning to the condensate phase, thus affecting condensation and higher-order structuring in macromolecular assemblies, including chromatin [21]. In eukaryotes, H3K9 methylation marks constitutive heterochromatin. Multivalent H3K9me3–chromodomain engagement triggers LLPS, through which constitutive heterochromatin is compartmentalized [47].

In addition, the transcription-initiation machinery and splicing machinery can form different phase-separated condensates. Phosphorylation of the RNA polymerase II (Pol II) C-terminal domain regulates the incorporation of Pol II into different phase-separated condensates. The hypophosphorylated C-terminal domain of Pol II is incorporated into mediator condensates, whereas the hyperphosphorylated C-terminal domain is preferentially incorporated into condensates that are formed by splicing factors. Phase separation promotes more efficient phosphorylation of the C-terminal domain of RNA polymerase II by CDK9, allowing for better transcriptional elongation [48]. These results suggest phosphorylation of the Pol II C-terminal domain as a mechanism that regulates a switch between transcriptional and splicing condensates [49].

## 3. Phase Separation in DNA Repair and Transcriptional Regulation

**Figure 2:**
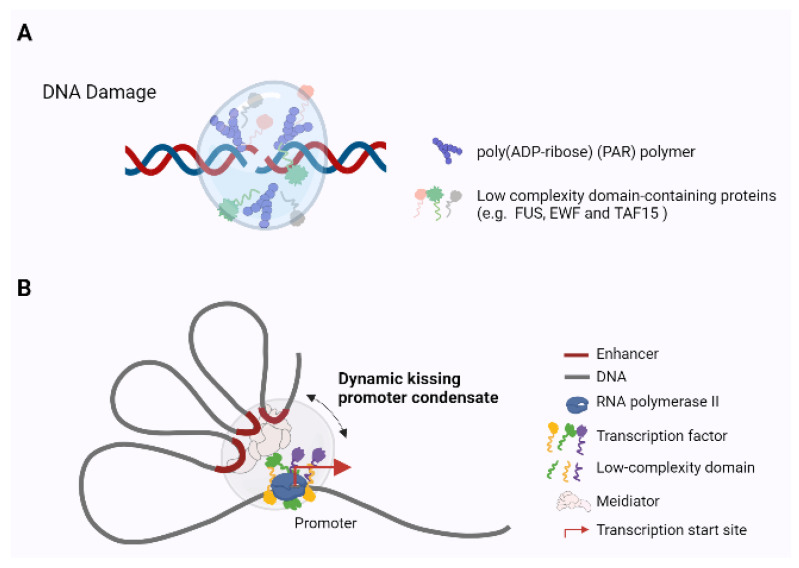
Phase separation in DNA repair and transcriptional regulation. (**A**) The role of phase separation in DNA damage repair. PAR chains, through phase separation, promote the transient and fully reversible assembly of various disordered proteins (such as FUS, EWF, and TAF15) at DNA break sites. (**B**) Phase separation in transcription-dependent chromatin condensate. The large cluster of mediators on the enhancer transiently kiss the transcriptional apparatus on promoter by phase separation, recruiting POLII to facilitate transcription initiation. In addition, TF LCDs interact to form local high-concentration hubs at both synthetic DNA arrays and endogenous genomic loci, which stabilize DNA binding, recruit RNA Pol II, and activate transcription.

During DNA repair and transcription in eukaryotes, chromatin modulates the access of regulatory factors to the genetic material. Several studies suggest that phase separation may help coordinate DNA repair and improve the efficiency of transcription (Figure 2).

### 3.1. Phase Separation in DNA Repair

In response to DNA damage, poly (ADP-ribose) polymerase 1 (PARP1) functions as a DNA damage sensor that rapidly recognizes and binds damaged DNA sites and recruits proteins to initiate DNA damage repair. During this process, PARP1 catalyzes the transfer of the polymeric ADPr unit from NAD+ to form the poly (ADP-ribose) (PAR) polymer [50]. The nucleic acid-like PAR polymer also plays an important role in the early events of the DNA damage response. PAR, through phase separation, promotes the transient and fully reversible assembly of various IDPs (such as FUS, EWF, and TAF15) at DNA break sites [51] (Figure 2A). The low-complexity domains (LCDs) of IDPs, including the positive charges in RGG repeats, are indeed necessary for electrostatic PAR interaction, and more PAR-responsive IDPs are yet to be discovered. This result suggests that PAR-driven phase separation may help coordinate the earliest stages of DNA repair by acting as a transient interaction filter for genome caretakers. In addition, phase separation of the DNA damage repair scaffold protein 53BP1 integrates localized DNA damage recognition and repair factor assembly with global p53-dependent gene activation and cell fate decisions [52]. Notably, PAR-mediated phase separation precedes the accumulation of 53BP1, which further suggests that these two protein assemblages do not intermix well and represent distinct, spatiotemporally separate entities at various stages of DNA damage [51,52].

### 3.2. Phase Separation in Transcriptional Regulation

During transcription, mediator and RNA polymerase II (Pol II) are colocalized in stable clusters that associate with chromatin in transcription-dependent condensates. Dynamic interactions between mediator clusters and motifs have been directly observed using two-color, three-dimensional (3D) live-cell imaging, supporting the dynamic “kissing” model, in which distal mediator clusters colocalize with genes only at certain time points (Figure 2B) [53]. In this model, large clusters of mediators on enhancers transiently “kiss” the transcriptional apparatus on the promoter, with some implications for the gene control machinery. The presence of large mediator clusters on some enhancers may allow mediator condensates on multiple gene promoters to simultaneously contact multiple gene promoters in the transcriptional apparatus [53]. Many eukaryotic transcription factors (TFs) contain intrinsically disordered low-complexity sequence domains. Single-molecule live-cell imaging has revealed that TF LCDs interact to form local high-concentration hubs at both synthetic DNA arrays and endogenous genomic loci, which stabilizes DNA binding with recruitment of RNA Pol II and subsequent transcriptional activate [54]. Prospero (Pros)/Prox1 is an evolutionarily conserved homeobox transcription factor that plays a role in promoting terminal differentiation. Pros is retained at the mitotic chromosomes of neural precursors via LLPS, where it recruits and condensates HP1, driving H3K9me3+ heterochromatin domain expansion and terminal neuronal differentiation [55].

## 4. Phase Separation in Mitotic Cell Cycle

**Figure 3:**
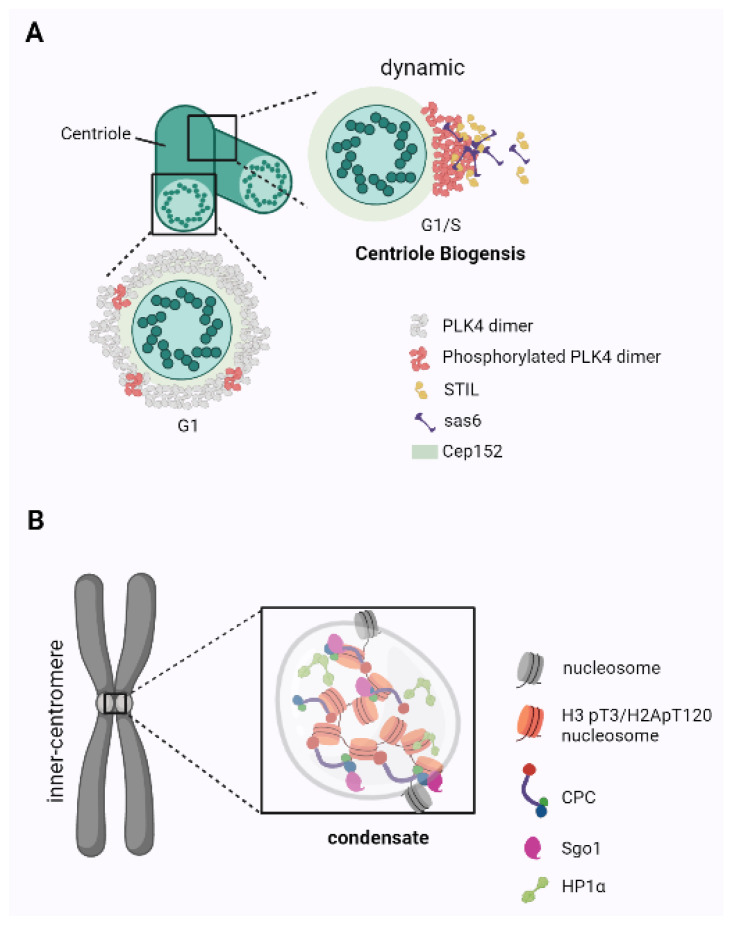
Regulation of mitotic chromosome behavior by phase separation. (**A**) Role of plk4 in the centriole biogenesis. In G1, Plk4 exhibits a ring-like state around the Cep152 scaffold. During G1/S, Plk4 exhibits a dot state at the future procentriole assembly site. Phosphorylated PLK4 promotes centriole genesis by phase-separated recruitment of STIL and Sas6 to the assembly site. (**B**) Localization of the CPC to the inner-centromere driven by CPC phase separation. CPC is recruited to inner-centromeres by multivalent interactions with Histone H3pT3, Sgo1, and HP1α.

In the majority of eukaryotic species, somatic cell proliferation occurs via dynamic mitosis, but until now, the mechanisms behind many chromosome-related events, such as checkpoint regulation, maintenance of nuclear state after the disappearance of the nuclear membrane, biogenesis of centrosome, formation of heterochromatin around the centromere, etc., have not been clarified. Here, we will summarize the role of phase separation in mitosis (Figure 3).

### 4.1. Phase Separation in Regulating Centriole Biogenesis

Centrosomes are the major microtubule-organizing centers in most animal cells and consist of a pair of centrioles surrounded by pericentriolar material (PCM). The formation of centrioles is essential for centrosome replication and must be closely coordinated with cell cycle progression. Studying the mechanisms regulating centriole biogenesis is essential for exploring the mechanism of chromosome segregation. Centriole replication is initiated by the assembly of a pre-centriole in the late G1/early S phase, in which the timely activation of Polo-like kinase 4 (Plk4) is central to the induction of centriole biogenesis [56,57] (Figure 3A). Early in G1, Plk4 exhibits a ring-like state around the Cep152 scaffold, followed by a dot state at the future procentriole assembly site, which is due to a dynamic phase separation process [58,59,60]. The autophosphorylation of the Plk4 non-catalytic cryptic polyphenylene box is a prerequisite for phase separation and the generation of nanoscale spherical condensates, which results in the recruitment of key centrosome components, such as STIL and Sas6, to the assembly site [61]. Interestingly, phase separation is not related to Plk4′s kinase activity; instead, these condensates form only in the presence of phosphorylated Plk4 [62].

### 4.2. Phase Separation in Chromosome Segregation

During mitosis, the maintenance of cohesion in the centromere chromatin region between sister chromatids is necessary for chromosome segregation [63]. During this time, the chromosomal passenger complex (CPC) abundantly condenses and localizes to the inner centromere, followed by the self-activation of Aurora-B kinase, and chromosomes begin to segregate [64] (Figure 3B). The CPC, which undergoes phase separation, not only drives its localization to the inner-centromere, but also corrects the connection between the centromere and microtubules and maintains a spindle checkpoint through multivalent interactions with phosphorylated histone H3 on Thr-3 (H3T3ph), sgo1 [6]. The C-terminal chromoshadow domain mediates HP1 dimerization and interaction with CPC [65]. Aurora B kinase regulates this interaction during mitosis by phosphorylating the adjacent serine 10 residue (H3S10ph), thereby releasing HP1 from chromatin. Therefore, CPC prevents the binding of HP1α and H3K9me3 and directly recruits HP1α to the inter centromere [66]. At this time, the NDR1-mediated hinge-specific phosphorylation of HP1a facilitates Sgo1 binding to centromeres directly or indirectly [67], which further strengthens the phase separation of CPC. These results suggest that the phase separation properties of HP1α and CPC jointly control the formation of heterochromatin around the centromere.

The control of cell entry into various phase nodes is closely related to local microenvironment regulation. During cell division, biomacromolecular components, such as transcription factors, are affected by local microenvironmental changes; quantitative changes trigger qualitative changes, resulting in independent membrane-free compartmentalized activities, many of which can be explained by phase separation. The establishment and stabilization of the dynamic liquid phase regulate the normal operation of the cell cycle and ensure the normal progress of mitosis. Overall, these suggest that phase separation plays an essential role in the regulation of cell mitosis.

## 5. Phase Separation in Meiotic Cell Cycle

Meiosis is a specialized cell division process that generates haploids from diploid germ cells. A series of events occur during this period, including homologous chromosome pairing, formation of programmed DNA double-strand breaks (DSBs), and homologous recombination, resulting in crossover formation, SC assembly, and chromosome remodeling. Defects in any of these steps can cause errors in meiotic chromosome segregation, which leads to the development of chromosome diseases, such as Down’s syndrome and Klinefelter syndrome. Therefore, understanding the mechanisms regulating the precise segregation of meiotic chromosomes is of great importance for reproductive health. There are still many meiotic phenomena that have not been fully explained. It may be possible to draw reasonable conclusions based on the mechanism of phase separation, and studies are now increasingly seeking breakthroughs in this area (Figure 3).

### 5.1. Homologous Chromosome Pairing

**Figure 4:**
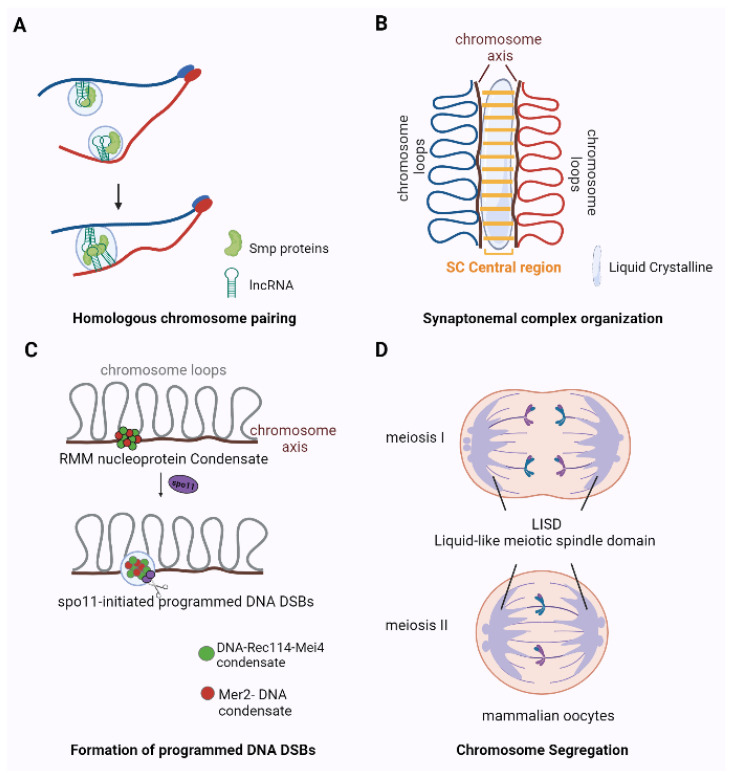
The role of phase separation in meiosis. (**A**) RNA–protein complexes promote the pairing of homologous chromosomes. Blue and red lines represent a set of homologous chromosomes held together at the telomeres. Homologous droplets (Smp proteins and IncRNA) fuse together, facilitating the pairing of homologous chromosomes. (**B**) Phase separation drives SC assembly. Weak interactions between SC central region proteins drive SC assembly, endowing the SC a liquid-crystalline property. (**C**) DNA-driven condensation assembles meiotic DSB formation machinery. Rec114-Mei4 and Mer2 independently condense with DNA into nucleoprotein clusters and then recruit Spo11 to induce DSB formation by phase-separated systems. (**D**) Liquid-like meiotic spindle domain (LISD) during meiosis I and meiosis II in mammalian oocytes. LISD promotes meiotic spindle assembly by selectively concentrating multiple microtubule regulatory factors and allows them to diffuse rapidly within the spindle volume.

The pairing of homologous chromosomes in meiosis is essential for sexual reproduction. In *Schizosaccharomyces pombe*, the *sme2* gene encodes a meiosis-specific 1500 nt long noncoding RNA (lncRNA) that accumulates at *sme2* chromosomal loci and mediates homologous recognition and pairing in meiosis [68]. Conserved RNA-binding Smp protein factors mediate phase separation at lncRNA transcription sites. The fusion of lncRNA–Smp droplets that occur at specific chromosomal loci mediate the recognition and subsequent pairing of homologous chromosomes [7] (Figure 4A). In addition, based on calculations and simulation, the Tartaglia team formulated the hypothesis that the lncRNA Xist uses phase separation to recruit repressive protein complexes to chromatin, which drives X chromosome inactivation [69].

### 5.2. Synaptonemal Complex Organization

During meiosis, paired homologous chromosomes are stabilized by a protein macromolecular structure called the synaptonemal complex (SC). Although the SC has a highly organized structure, it is not static, and its components keep coming on and off from the chromosome during early prophase. This dynamic behavior is thought to be essential for its function in regulating meiotic recombination and crossover formation [70,71,72]. The SC has been reported to exhibit liquid crystalline properties that may underlie crossover regulation [73,74]. SC central region proteins typically contain both coiled-coil structures and IDRs. While the former is known to mediate interactions between SC components [75,76,77], the role of IDRs in SC assembly and dynamics remains a mystery. The IDRs in SC proteins are rich in charge-interacting elements, which are involved in multivalent weak interactions between SC assembly units that give SC its liquid crystalline properties in some organisms (Figure 4B). The presence of charge-interacting elements in SC proteins suggests a type of driving force for SC assembly via phase separation [8]. Other types of interactions may also exist, such as hydrophobic interactions. Moreover, protein modifications, such as phosphorylation and N-terminal acetylation may affect various types of interactions between SC components and regulate SC formation [78].

### 5.3. Formation of Programmed DNA DSBs

The accurate segregation of chromosomes during meiosis is critical for genome stability. It relies on homologous recombination initiated by the Spo11 protein that introduces DNA DSBs. The formation of DSBs is regulated by protein assemblies and is associated with the formation of large-scale chromosome structures. In *Saccharomyces cerevisiae*, RMM proteins consisting of Rec114, Mei4, and Mer2 are essential conserved components of the DSB machinery. Rec114, Mei4, and Mer2 independently condense with DNA into nucleoprotein clusters and then recruit Spo11 to induce DSB formation through phase-separated systems [9] (Figure 4C). In addition, nucleoprotein clusters may regulate DSB repair by controlling the DSB number, location, and timing and coordinate DSB formation [9]. The formation of DNA damage response foci requires damage-induced long non-coding RNA synthesized at DSBs by RNA polymerase II. Damage-induced long non-coding RNA promotes the repair of DSBs by driving DDR protein recruitment to damage sites in response to DNA damage through phase separation [79].

### 5.4. Spindle Assembly and Chromosome Segregation

The spindle apparatus is composed of microtubules (MTs), a spindle matrix, and MT-associated proteins, which are indispensable in mitosis and meiosis. As a conserved spindle matrix protein found in both vertebrates and invertebrates, phase transition of BuGZ concentration of tubulin and promotion of MT polymerization, thus further promoting spindle matrix assembly and functional spindle formation [80]. Centrosomes are major MT-organizing centers that are important for spindle assembly, polarity establishment, and cell division [81]. In *C. elegans*, the PCM scaffold protein SPD5 undergoes phase separation and recruits microtubule polymerase ZYG-9 and the microtubule-stabilizing protein TPXL-1 to promote MT nucleation [81]. Centrosomin, the functional ortholog protein of SPD5 in *Drosophila*, plays a similar role in centrosome assembly [82]. In *Xenopus* eggs, electrostatic residues at the N-terminal 1-480aa of TPX2 interacts with tubulin via LLPS to form a local reservoir on preexisting microtubules, which may be necessary to efficiently promote branching microtubule nucleation in an all-or-none manner [83]. Different from somatic cells and male germ cells, mammalian oocytes lack a centrosome and form a unique liquid-like meiotic spindle domain (LISD) during meiosis (Figure 4D). The LISD promotes meiotic spindle assembly by selectively concentrating multiple microtubule regulatory factors and allowing them to diffuse rapidly within the spindle volume [10]. Therefore, it is clear that the phase separation of MT regulator proteins regulates spindle MT dynamics, but the mechanisms involved in spindle assembly are not the same in different species.

## 6. Conclusions and Prospects

In recent years, phase separation has gradually become a topic of interest in the biomedical field. An increasing number of studies have confirmed the close connection between phase separation and the occurrence of membraneless organelles, and phase separation also plays an important role during reproductive development. Herein, we reviewed how phase separation regulates chromatin and chromosome organization and controls mitotic and meiotic chromosome behavior. On the one hand, it is based on the self-regulation of the chromatin assembly unit, while on the other hand, chromatin-associated proteins actively participate in chromatin assembly. Phase separation can be used to explain many regulatory mechanisms of cell division-related biological processes in the nucleus. Nevertheless, not all phase separation processes are necessarily linked to physiological functions. A large number of experiments are still needed to provide definitive conclusions. However, there are studies that have made some promising progress from the perspective of DNA and RNA involvement in phase transitions. LncRNA assists in protein localization and directly or indirectly regulates protein phase separation; differences in nucleotide length and base sequence also have an effect on DNA condensation, and protein-independent homology recognition between dsDNA can improve the efficiency of meiotic homologous recombination [13].

The chemical microenvironment synergistically changes with phase separation. Changes in in vitro solution conditions, such as the temperature, salt concentration, and cation level, directly affect protein phase separation [84,85]. Changes in physiological conditions lead to changes in the level of protein condensation, which is key to triggering phase separation. The study of the mechanism by which cells regulate these microenvironmental changes is undoubtedly of great significance for studying the key events in cell division. In addition, it has been found that the phase separation state of many macromolecules, such as proteins, gradually changes from the liquid phase to the solid phase over time [86,87], yet the mechanism by which cells control such a transition remains elusive. Moreover, research on diseases associated with protein aggregation may be explained by phase separation processes, such as the aggregation of protein mutations in neurodegenerative diseases [88,89], which will be a key research direction in the future. In addition to direct experimental evidence, computational modelling could be used to better understand biological phase separation. Combining experimental systems with theoretical frameworks, as conducted with the Corelet system, will help elucidate the underlying biophysical mechanisms by which intracellular phase transitions are controlled in cells, both globally and locally [90]. Many phase separation-related databases have been established [91,92], and it can be predicted that more studies will use bioinformatics to simulate and predict phase behavior in the future.

Our understanding of the dynamic regulation of biological processes in cells has been revolutionized. However, there are still many questions that remain to be addressed. How is the composition of biomolecular condensates in cells specified? What mechanism dynamically regulates multivalent interactions between proteins? How does phase separation contribute to mitosis and meiosis and initiate different regulatory mechanisms? The current research tools mainly include isothermal titration calorimetry [93], fluorescence recovery after photobleaching [3], fluorescence resonance energy transfer [13], atomistic simulations [14,91,92], and polymer simulations [15]. Due to the nature of diffraction-limited intracellular biomacromolecular condensates and the limitations of experimental methods, it is difficult to directly visualize phase separation in vivo. There is an urgent need to develop new techniques to resolve the fine structure of phase separation. More phase separation regulation mechanisms will eventually be revealed with the vigorous development of emerging technologies in the study of biological phenomena at the microscopic level.
